# Mixed-Dimensional Nanowires/Nanosheet Heterojunction of GaSb/Bi_2_O_2_Se for Self-Powered Near-Infrared Photodetection and Photocommunication

**DOI:** 10.1007/s40820-025-01793-2

**Published:** 2025-06-03

**Authors:** Guangcan Wang, Zixu Sa, Zeqi Zang, Pengsheng Li, Mingxu Wang, Bowen Yang, Xiaoyue Wang, Yanxue Yin, Zai-xing Yang

**Affiliations:** School of Physics, State Key Laboratory of Crystal Materials, Shandong University, Jinan, 250100 People’s Republic of China

**Keywords:** Near-infrared photodetection, Self-powered photodetection, Mixed-dimensional heterojunction, GaSb nanowire, Bi_2_O_2_Se nanosheet

## Abstract

With high surface-to-volume ratio, the abundant surface states and high carrier concentration are challenging the near-infrared photodetection behaviors of narrow band gap semiconductors nanowires. In this study, the narrow band gap semiconductor of Bi_2_O_2_Se nanosheets (NSs) is adopted to construct mixed-dimensional heterojunctions with GaSb nanowires (NWs) for demonstrating the impressive self-powered NIR photodetection. Benefiting from the built-in electric field of ~ 140 meV, the as-constructed NW/NS mixed-dimensional heterojunction self-powered photodetector shows the low dark current of 0.07 pA, high *I*_light_/*I*_dark_ ratio of 82 and fast response times of < 2/2 ms at room temperature. The self-powered photodetector performance can be further enhanced by fabricating the NW array/NS mixed-dimensional heterojunction by using a contact printing technique. The excellent photodetection performance promises the as-constructed NW/NS mixed-dimensional heterojunction self-powered photodetector in imaging and photocommunication.

**Supplementary Information:**

The online version contains supplementary material available at 10.1007/s40820-025-01793-2.

## Highlights

- The mixed-dimensional type II heterojunction of GaSb nanowires (NWs) and Bi_2_O_2_Se nanosheets (NSs) with a built-in electric field of ~ 140 meV is successfully constructed.
- As-fabricated NW/NS and NW array/NS mixed-dimensional heterojunction photodetectors exhibit as-expected high-performance self-powered photodetection behaviors, including ultralow I_dark_ (0.07 and 0.08 pA), superior I_light_/I_dark_ ratios (82 and 182) and ultrafast photoresponse (< 2/2 and 6/4 ms).
- As-fabricated NW array/NS mixed-dimensional heterojunction self-powered photodetector promises the future imaging and photocommunication.

**Supplementary Information:**

The online version contains supplementary material available at 10.1007/s40820-025-01793-2.

## Graphical abstract

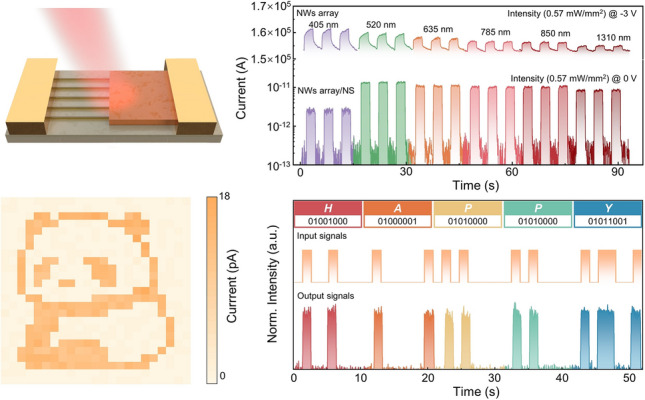

## Introduction

Near-infrared (NIR) photodetector has attracted much more attention in remote sensing, medical imaging and environmental monitoring, due to its ability to penetrate biological tissues and the atmosphere [1–12]. With the moderate narrow band gap of 0.72 eV, the highest hole mobility of 850 cm^2^ V^−1^ s^−1^ among III–V semiconductors and the broad spectral absorption ability, GaSb nanowires (NWs) have been utilized for near-infrared photodetection in the past decade, which is in forms of photoconductor, field-effect phototransistor and Schottky photodiode [13–17]. With high surface-to-volume ratio, the abundant surface states and high carrier concentration are challenging the NIR photodetection behaviors of GaSb NWs [17–19]. First of all, the surface state will act as the carrier traps, resulting in the bias stress instability of GaSb NW NIR photodetectors [15]. Furthermore, the surface state will cause the famous Fermi level pinning, resulting in the inability to modulate the dark current (I_dark_) of the NIR photodetector through the metal/semiconductor contact barrier [13, 20]. At the same time, the high carrier concentration will cause the NIR photodetector to suffer from a large I_dark_, resulting in a low I_light_/I_dark_ [21].

To date, the surface passivation, van der Waals integration and construction of heterojunction have been developed to optimize the NIR photodetection behaviors of low-dimensional semiconductors [13, 20, 22–24]. Among them, the construction of heterojunction is a popular and meaningful approach, because the built-in electric field and depletion region would be introduced at the semiconductor–semiconductor heterointerfaces by designing reasonably the energy band engineering, benefiting to the quick separation and collection of photogenerated electron–hole pairs, which facilitates the faster photoresponse speed [25–31]. Furthermore, the built-in electric field and depletion region benefit to the success construction of self-powered photodetector, which operates effectively without an external energy source, hold great potential applications in Internet of Things and low power dissipation. At the same time, the I_dark_ will be significantly suppressed and photosensitivity is effectively enhanced due to the barrier at the interfaces and the absence of an external bias, benefiting to the smart photodetection of NIR light [32].

It is worth pointing out that the type II semiconductor heterostructure promotes the separation of photogenerated carriers by directing electrons and holes across the interface in opposite directions [33–35]. This property makes the type II heterojunctions ideal candidates for ultrasensitive and self-powered photodetectors [36–41]. With narrow band gap of 0.8 eV, Bi_2_O_2_Se nanosheets (NSs) are adopted to construct mixed-dimensional type II heterojunctions with GaSb NWs for demonstrating the impressive self-powered NIR photodetection, optical imaging and photocommunication in this work [42, 43]. Benefiting from the built-in electric field, the as-fabricated NW/NS heterojunction photodetector exhibits excellent self-powered photodetection performance, that is, the *I*_dark_ is as low as 0.07 pA, the *I*_light_/*I*_dark_ ratio is as high as 82, and the response times are as fast as < 2/2 ms, which significantly outperform the NW and NS photodetectors. Furthermore, the self-powered photodetection performance is further improved by constructing NW array/NS heterojunction photodetector. The fabricated NW array/NS heterojunction self-powered photodetector exhibits low *I*_dark_ of 0.08 pA, high *I*_light_/*I*_dark_ ratio of 182 and fast optical response times of 6/4 ms. In the end, the fabricated NW array/NS heterojunction also enables self-powered imaging and photocommunication capabilities. These results demonstrate that the construction of GaSb NW/Bi_2_O_2_Se NS mixed-dimensional heterostructures promises the next-generation high-performance self-powered NIR photodetection.

## Experimental Section

### **Growth of GaSb NWs and Bi**_**2**_**O**_**2**_**Se NSs**

GaSb NWs are prepared by using a surfactant-assisted CVD method in a dual-zone horizontal tube furnace [44]. High-purity GaSb powder (99.999%) is placed in the upstream zone, a Si/SiO_2_ growth substrate coated with a 1-nm Pd catalyst is positioned in the downstream zone, and sulfur powder (99.99%) is placed between the two zones. The precursor vapor from the upstream zone is carried to the downstream zone by using hydrogen gas (99.999% purity). The CVD system is evacuated to 6 × 10^–3^ Torr and purged with 200 sccm of H_2_ for 30 min prior to heating. Upon reaching the designated time, the source and substrate heaters are turned off simultaneously, allowing the system to cool to room temperature under a hydrogen flow.

The Bi_2_O_2_Se NSs are prepared on the mica by CVD method in a dual-zone horizontal tube furnace [45]. Source powder of Bi_2_O_3_ (0.1 g, 99.99%) is placed in the heating center of the first zone, and Bi_2_Se_3_ powder (0.01 g, 99.99%) is placed 6 cm upstream. The freshly cleaved mica is placed 6 cm upstream of the heating center in the second zone. Argon (200 sccm, 99.999%) with a tube pressure maintained at 60 Torr is employed as the carrier gas to transport the precursors onto a mica surface for the growth of Bi_2_O_2_Se NSs. The temperatures in the two zones are set as 690 and 560 °C for 40 min to obtain the Bi_2_O_2_Se NSs. After the growth process, the system is subsequently cooled to room temperature while maintaining a flow of argon gas.

### **Fabrication of GaSb/Bi**_**2**_**O**_**2**_**Se Mixed-Dimensional Heterojunctions Photodetectors**

First, for a single GaSb NW photodetector, the as-prepared NWs are suspended in an ethanol solution using ultrasonication and subsequently transferred onto Si substrates with a 50-nm SiO_2_ layer via drop casting. For NW array photodetectors, the ordered GaSb NWs are transferred onto Si substrates with a 50-nm SiO_2_ layer using a contact printing technique. Subsequently, the prepared Bi_2_O_2_Se NS is transferred from mica onto a SiO_2_/Si substrate with GaSb NW or NW array using a wet transfer method, supported by polymethyl methacrylate (PMMA) in a 1% dilute HF solution and finally cleaned with acetone. Finally, the contact electrodes are patterned using standard electron beam lithography, followed by the deposition of 50-nm Ni metal electrodes through a thermal evaporation system.

### **Characterization of GaSb/Bi**_**2**_**O**_**2**_**Se Mixed-Dimensional Heterojunctions and Photodetectors**

The morphology of the as-prepared GaSb NWs and Bi_2_O_2_Se NSs is characterized using a microscope (Olympus microscope BX53 M) and scanning electron microscopy (SEM, KYKY-EM6900). A scanning probe microscope (Horiba Bruker Multimode 8) equipped with atomic force microscopy (AFM) and Kelvin probe force microscopy (KPFM) modules is used to study the material thickness and surface potential. The photodetection performance of the as-prepared photodetectors is measured using an Agilent B1500A semiconductor analyzer connected to a probe station at room temperature. Diode lasers are used as light sources for photodetection measurements.

## Results and Discussion

### Design and Construction of Mixed-dimensional NW/NS Heterojunctions

**Fig. 1:**
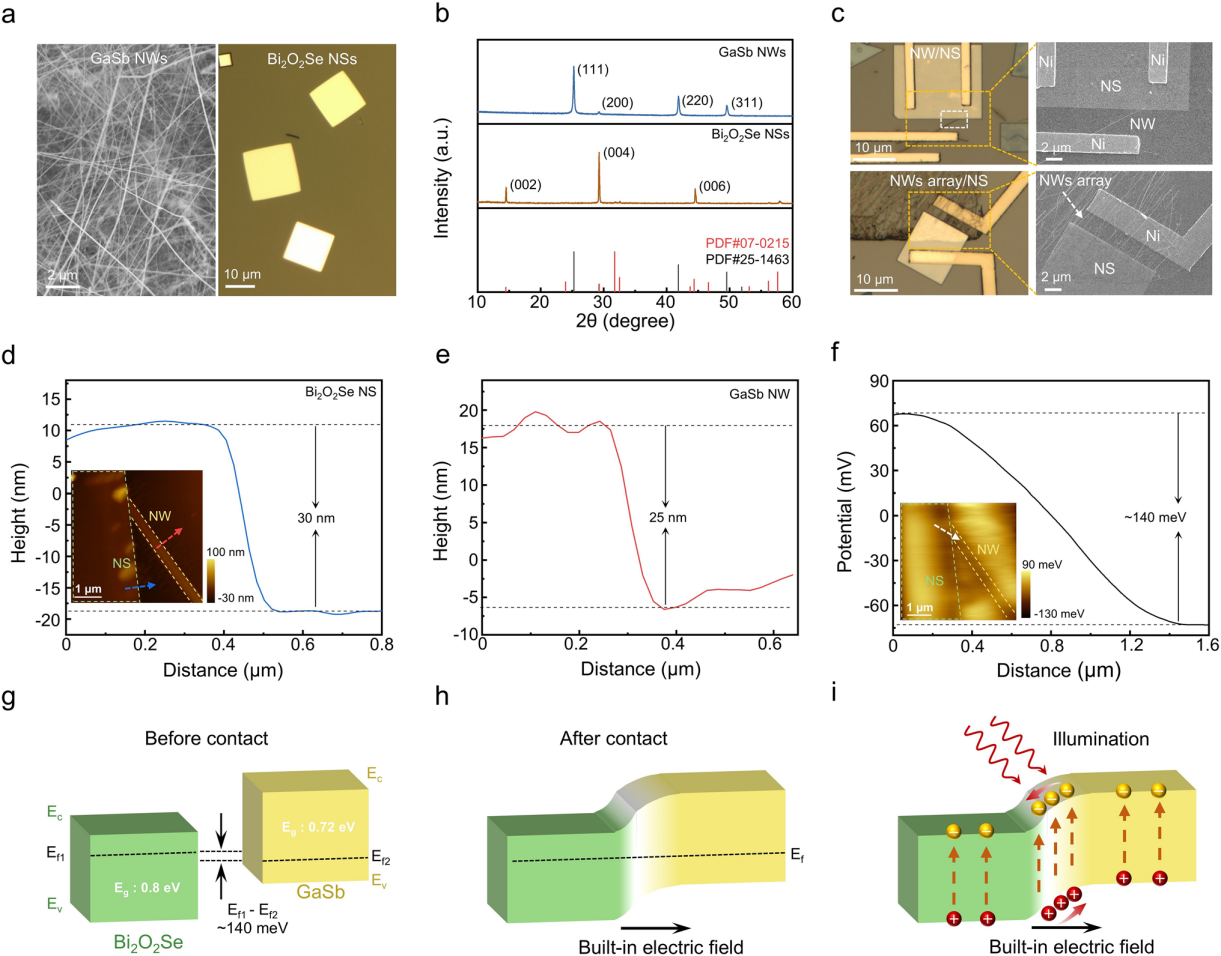
Construction of GaSb/Bi_2_O_2_Se mixed-dimensional NW/NS heterojunctions for self-powered photodetection. **a, b** SEM, OM images and XRD patterns of GaSb NWs and Bi_2_O_2_Se NSs, respectively. **c** OM and SEM images of NW/NS and NW array/NS heterojunction, respectively. **d, e** Height profiles of Bi_2_O_2_Se NS and GaSb NW. Inset is the AFM image of GaSb/Bi_2_O_2_Se NW/NS heterojunction. **f** Surface potential difference profile of GaSb/Bi_2_O_2_Se NW/NS heterojunction. **g–i** Schematic of the self-powered GaSb/Bi_2_O_2_Se NW/NS heterojunction photodetector (the E_c_, E_v_ and E_f_ are the conduction band minimum, valance band maximum and Fermi level, respectively)

GaSb NWs and Bi_2_O_2_Se NSs are prepared by chemical vapor deposition (CVD) method in a dual-zone tube furnace. The detailed experimental procedures are described in “Methods” section. SEM image in Fig. 1a shows that the as-prepared GaSb NWs have uniform diameter and smooth surface. Optical microscopy (OM) image reveals that the as-prepared Bi_2_O_2_Se NSs exhibit regular square morphology with size up to approximately 20 μm. X-ray diffraction (XRD) patterns (Fig. 1b) confirm that the GaSb NWs have a pure zinc-blende crystal structure (JCPDS card No. 07–0215), while Bi_2_O_2_Se NSs exhibit tetragonal crystal phase (JCPDS card No. 25–1463). The as-prepared GaSb NWs and Bi_2_O_2_Se NSs are then adopted to construct mixed-dimensional heterojunctions by PMMA-assisted wet transfer method, as reported in the literature [42]. Figure 1c displays the OM and SME images of as-constructed GaSb/Bi_2_O_2_Se NW/NS and NW array/NS mixed-dimensional heterojunctions, demonstrating the high-quality contact between NWs and NSs. AFM is then employed to measure the diameter of GaSb NW and the thickness of Bi_2_O_2_Se NS in the as-constructed GaSb/Bi_2_O_2_Se NW/NS mixed-dimensional heterojunction, as shown in Fig. 1d, e. As a result, the diameter is 25 nm and the thickness is 30 nm. Furthermore, the Kelvin probe force microscopy (KPFM) is adopted to measure the surface potentials of GaSb NW and Bi_2_O_2_Se NS, demonstrating the Fermi level difference. The measured area is as same as the AFM image in the inset of Fig. 1d. In this case, the potential curve is obtained from the cross of NW and NS, as depicted by the white line in the inset of Fig. 1f. As shown in Fig. 1f, Bi_2_O_2_Se NS exhibits a higher surface potential than GaSb NW, with a Fermi level difference of approximately 140 meV. The schematic of energy band alignment is then shown in Fig. 1g-i, benefiting to the explanation of photogenerated carrier transfer behavior at the heterostructure interface. As reported in the studies, the band gaps of GaSb NW and Bi_2_O_2_Se NS are approximately 0.72 and 0.8 eV, respectively [14, 20]. When GaSb and Bi_2_O_2_Se come into contact, the higher Fermi level of Bi_2_O_2_Se facilitates the transfer of electrons into GaSb, leading to the thermal equilibrium and unified Fermi level. At the same time, the band will bend at the interface, forming a useful type II heterojunction. It is worth pointing out that the built-in electric field and depletion region form at the interface, which directs from Bi_2_O_2_Se NS to GaSb NW. In this case, under the illumination with wavelength shorter than 1310 nm, the photogenerated electron–hole pairs will be separated and collected quickly without an external energy source, which facilitates the self-powered photodetection behaviors with faster photoresponse speed and reduced I_dark_. In summary, the as-constructed GaSb/Bi_2_O_2_Se mixed-dimensional heterojunctions promise the next-generation self-powered high-performance NIR photodetection.

### Photodetection Behaviors of Mixed-dimensional NW/NS Heterojunction

**Fig. 2:**
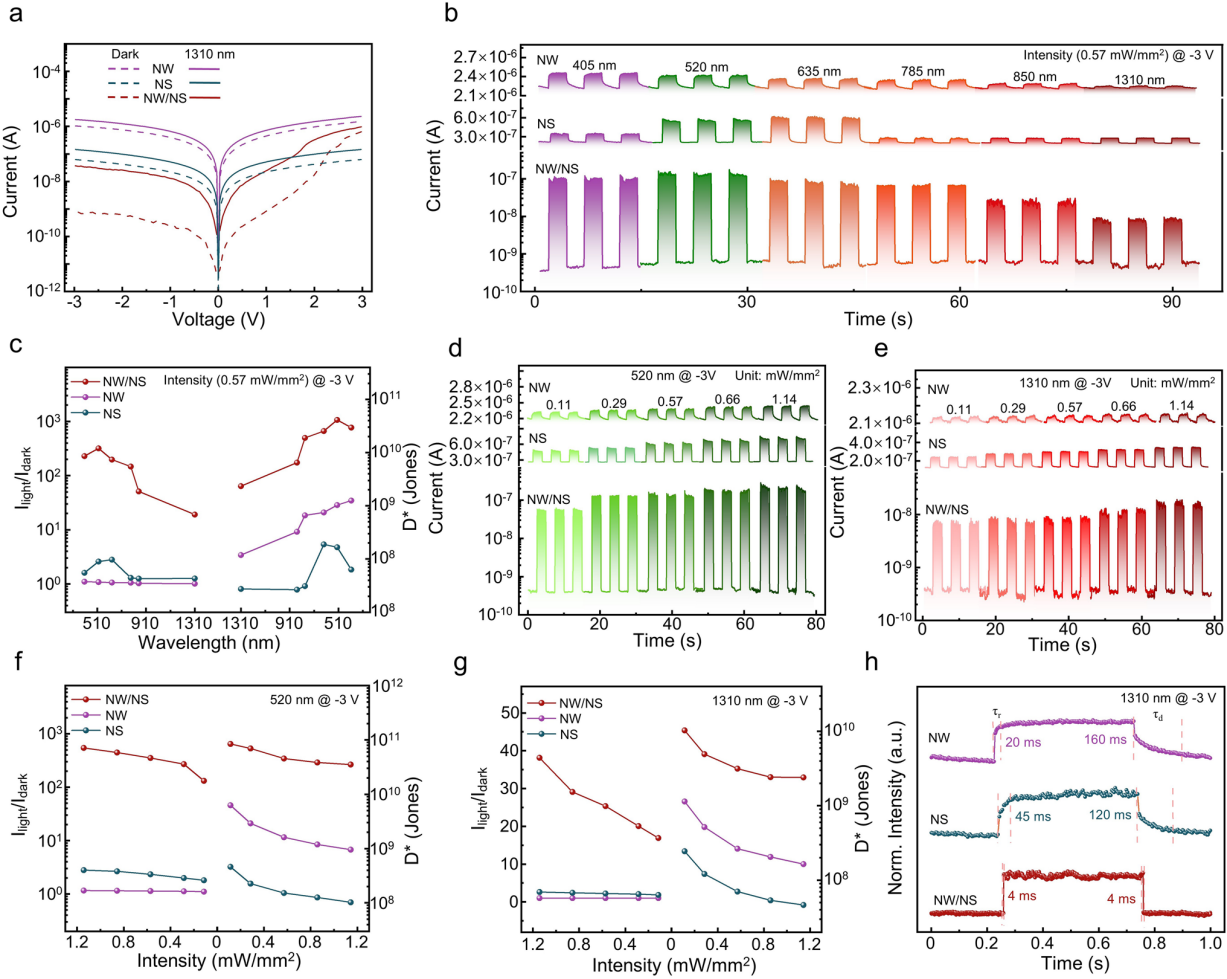
Photodetection behaviors of GaSb NW, Bi_2_O_2_Se NS and GaSb/Bi_2_O_2_Se mixed-dimensional NW/NS heterojunction.** a** I–V characteristics of the NW, NS and NW/NS mixed-dimensional heterojunction photodetectors in dark and under the illumination of 1310-nm laser with power intensity of 0.57 mW mm^−2^. **b** Wavelength-dependent temporal photoresponse of the NW, NS and NW/NS heterojunction photodetectors. **c** I_light_/I_dark_ ratio, D* and response time of the NW, NS and NW/NS heterojunction photodetectors under the illuminations of 405–1310-nm laser with power intensity of 0.57 mW mm^−2^. **d, e** I–t curves of the NW, NS and NW/NS heterojunction photodetectors under the illumination of 520- and 1310-nm lasers, respectively. **f, g** I_light_/I_dark_ ratio and D* of the NW, NS and NW/NS heterojunction photodetectors under the illumination of 405- and 1310-nm laser, respectively. **h** Response times of the NW, NS and NW/NS heterojunction photodetectors

After the success in constructing NW/NS heterojunction, the corresponding photodetection behaviors are then studied in Fig. 2. Figure 2a presents the I–V curves of NW, NS and NW/NS heterojunction photodetectors in dark and under the illumination of 1310-nm laser with an intensity of 0.57 mW mm^−2^. It is worth pointing out that both of the as-fabricated GaSb NW and Bi_2_O_2_Se NS photodetectors exhibit Ohmic contacts, which rules out the influence of the Schottky barrier for the heterostructure, as depicted in Fig. S1. Under −3 V bias, the NW/NS heterojunction photodetector shows the lowest *I*_dark_ of 0.5 nA and the highest *I*_light_/*I*_dark_ ratio of 20. Figure 2b shows the wavelength-dependent photoresponse of the three photodetectors under −3 V bias. Benefiting from the narrow band gaps of GaSb and Bi_2_O_2_Se, the as-fabricated NW, NS and NW/NS heterojunction photodetectors all exhibit broadband photodetection capability. Notably, the *I*_dark_ is significantly suppressed after constructing NW/NS heterojunction. As shown in Fig. 2c, as expected, due to the suppressed *I*_dark_, the *I*_light_/*I*_dark_ ratio of the heterojunction photodetector is improved across the wavelength range of 405–1310 nm. The *I*_light_/*I*_dark_ ratio of NW/NS heterojunction photodetector is substantially enhanced by factors of 294 and 122 compared to NW and NS photodetectors. Two critical parameters of responsivity (R) and detectivity (D*) are also optimized in Figs. S2 and 2c. *R* can be defined as *I*_ph_/(PA), and D* can be defined as RA^1/2^/(2*eI*_dark_)^1/2^, in which *P* is the incident power density, *A* is the effective irradiated area, e is the electronic charge, and I_ph_ is defined as *I*_light_−*I*_dark_ [46]. In this case, the values of *R* and D* are 2.3 × 10^3^, 3.2, 9.7 × 10^2^ A W^−1^ and 1.0 × 10^9^, 1.7 × 10^8^, 4.1 × 10^10^ Jones for NW, NS and NW/NS heterojunction photodetectors, respectively.

The intensity-dependent photoresponse of the three photodetectors under the illuminations of 520- and 1310-nm lasers is shown in Figs. 2d, e and S3. The I_light_ of all three devices increases linearly with the increase in the incident light intensity. Among them, the I_light_ of NW/NS heterojunction photodetector increases from 59.7 and 7.7 nA to 247.7 and 17.3 nA under the illuminations of 520- and 1310-nm lasers with the intensity increases from 0.11 to 1.14 mW mm^−2^. Figure 2f, g shows the intensity-dependent I_light_/I_dark_ ratio and D* of the three photodetectors. The *I*_light_/*I*_dark_ ratio increases with the increase in the laser intensity, while the D* decreases as the laser intensity increases. Notably, the NW/NS heterojunction photodetector achieves a maximum *I*_light_/*I*_dark_ ratio of 545 and 38, and a D* of 8.4 × 10^10^ and 1.0 × 10^10^ Jones, respectively, under the illuminations of 520 and 1310 nm, further demonstrating the decreased I_dark_ and excellent photosensitivity. The response time, including the rise time (*τ*_r_) and the decay time (*τ*_d_), is another critical parameter of photodetectors. It refers to the time required for photocurrent to rise from 10 to 90% or fall from 90 to 10% [47]. As shown in Fig. 2h, the NW/NS heterojunction photodetector achieves τ_r_/τ_d_ values of 4/4 ms, much shorter than the 20/160 and 45/120 ms of NW and NS photodetectors, respectively. In short, the as-constructed NW/NS mixed-dimensional heterojunction promises the next-generation high-performance photodetectors.

**Fig. 3:**
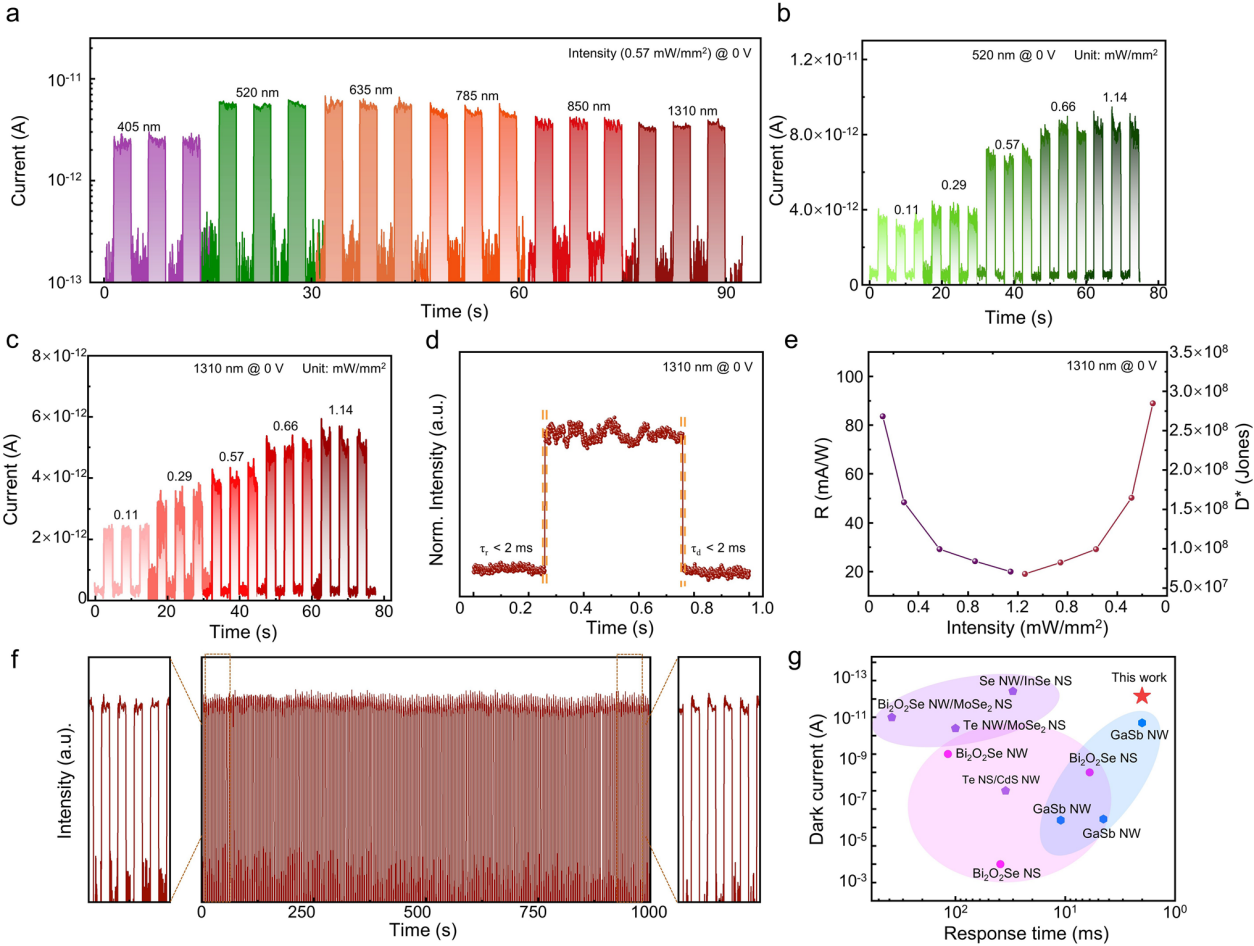
Self-powered NIR photodetection behaviors of GaSb/Bi_2_O_2_Se mixed-dimensional NW/NS heterojunction. **a** Wavelength-dependent photoresponse of the NW/NS heterojunction self-powered photodetector. **b, c** Intensity-dependent photoresponse under the illuminations of 520- and 1310-nm lasers. **d-f** Response time, R, D* and long-term stability of NW/NS heterojunction self-powered NIR photodetector. **g** I_dark_ and response time comparison between this work and other photodetectors previously reported in the studies, including GaSb NW, Bi_2_O_2_Se NS, mixed-dimensional heterojunctions. References to the selected work can be found in Table S1

The built-in electric field facilitates the self-powered NIR photodetection behaviors of NW/NS heterojunction photodetector, which are detailedly studied in Fig. 3. As shown in Fig. 3a, NW/NS heterojunction photodetector exhibits as-expected broadband photodetection capability under no external bias voltage, along with extremely low I_dark_ of 0.07 pA. The dark current fluctuation is caused by the resolution limitation of semiconductor analyzer. The self-powered photodetection behaviors of the NW/NS heterojunction photodetector are further studied under the illumination of 520- and 1310-nm lasers, as shown in Fig. 3b, c. The I_light_ increases from 3.8 and 2.5 to 9.5 and 5.9 pA under the illuminations of 520- and 1310-nm lasers with the intensity increases from 0.11 to 1.14 mW mm^−2^, while the *I*_dark_ is maintained at a level of 0.07 pA. The *I*_light_/*I*_dark_ ratio increases with the increase in the laser intensity, referring the superior photosensitivity of the NW/NS heterojunction. In self-powered mode, the photodetector exhibits *τ*_r_ and *τ*_d_ of < 2 ms each, as illustrated in Fig. 3d. The NIR photodetection performance of the NW/NS heterojunction photodetector is further evaluated by *R* and D*, as shown in Fig. 3e. Under the illumination of 1310 nm with power intensity of 0.11 mW mm^−2^, the *R* and D* reach 84 mA W^−1^ and 2.85 × 10^8^ Jones, respectively. Operation stability is a critical metric of photodetectors. Figure 3f exhibits the photoresponse of the NW/NS heterojunction self-powered photodetector over an operation time of 1000 s. No significant I_light_ attenuation demonstrates the excellent operational stability. The as-fabricated NW/NS heterojunction photodetector is compared with other photodetectors with similar material systems or configurations in Fig. 3g. Clearly, the as-fabricated NW/NS heterojunction photodetector outperforms most counterparts in terms of fast response speed and exceptionally low I_dark_. In summary, the NW/NS mixed-dimensional heterojunction photodetector exhibits outstanding self-powered NIR photodetection performance.

### Photodetection Behaviors of Mixed-dimensional NW Array/NS Heterojunction

**Fig. 4:**
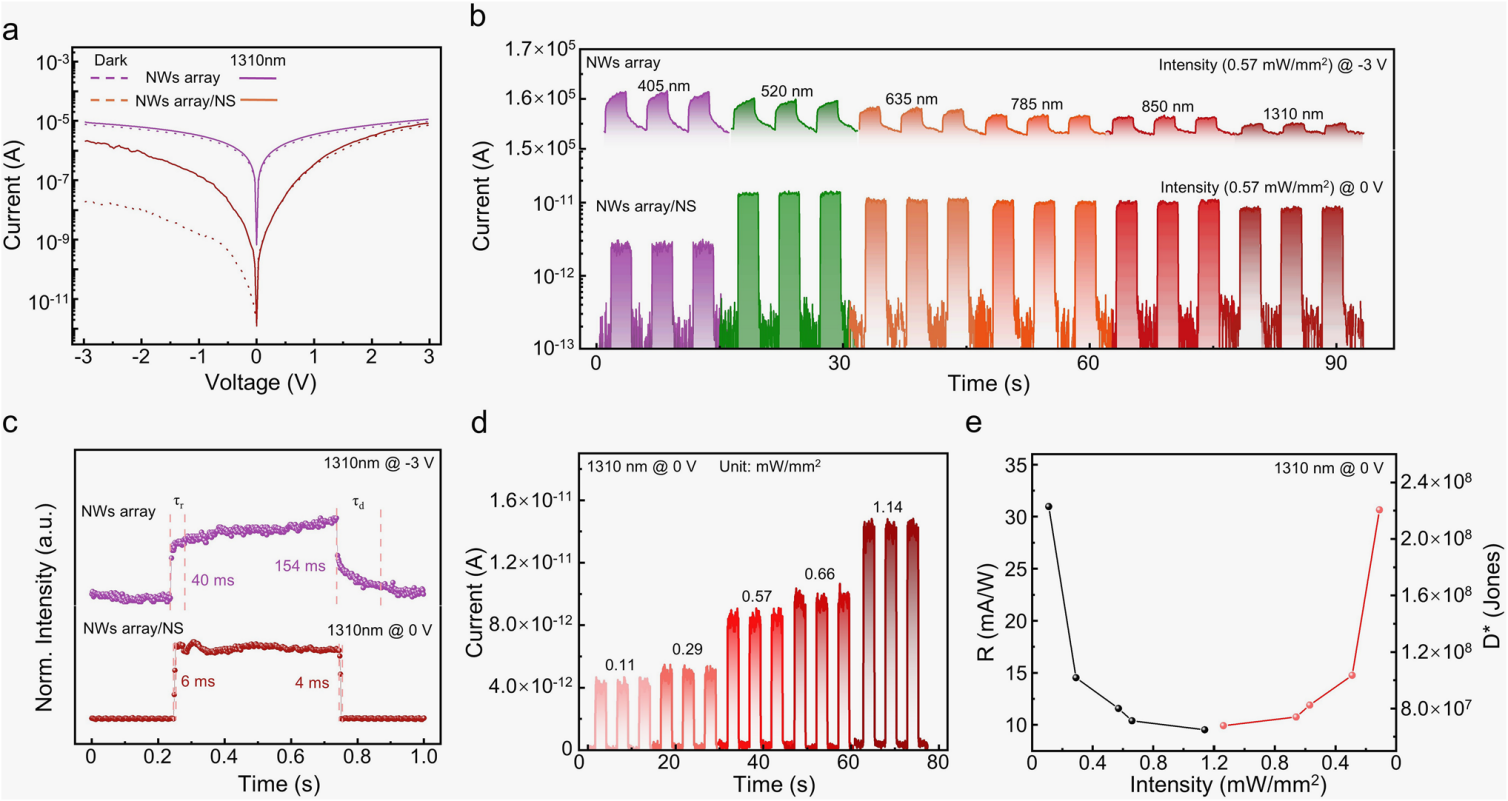
Photodetection behaviors of GaSb array and GaSb/Bi_2_O_2_Se mixed-dimensional NW array/NS heterojunction. **a** I–V characteristic curves of the NW array and NW array/NS heterojunction photodetectors in dark and under the illumination of 1310-nm laser (0.57 mW mm^−2^). **b** Wavelength-dependent temporal photoresponse of the NW array and NW array/NS heterojunction photodetector. **c** Response time of the NW array and NW array/NS heterojunction photodetectors under the illumination of 1310-nm laser. **d** Self-powered photodetection behaviors of NW array/NS mixed-dimensional heterojunction photodetector under the illumination of 1310-nm laser. **e** *R* and D* of the self-powered NW array/NS heterojunction photodetector versus the incident light (1310-nm) intensity

Ordered NW array has been considered promising candidates for the large-scale integration of optoelectronic devices [57, 58]. The ordered NW array is obtained by the reported contact printing technology [59, 60]. Then, the photodetection behaviors of NW array and the NW array/NS mixed-dimensional heterojunction photodetectors are studied in Fig. 4. Figure 4a exhibits the I–V curves of NW array and NW array/NS mixed-dimensional heterojunction photodetectors in dark and under the illumination of 1310-nm laser with an intensity of 0.57 mW mm^−2^. Under −3 V bias, the NW/NS heterojunction photodetector shows the lowest *I*_dark_ of 32 nA and the highest *I*_light_/*I*_dark_ ratio of 60. The *I*_dark_ of the NW array/NS heterojunction photodetector is significantly reduced, approximately 600 times lower than that of the NW array photodetector. The wavelength-dependent photoresponse of the NW array and NW array/NS heterojunction photodetectors is shown in Figs. 4b and S4a, further demonstrating the improved *I*_light_/*I*_dark_ ratio and decreased *I*_dark_ (0.08 pA). More importantly, the increased contact area between GaSb NWs and Bi_2_O_2_Se NS results in a larger photocurrent, which is twice as large as that of the NW/NS photodetector. The *τ*_r_ and *τ*_d_ of the NW array/NS heterojunction self-powered photodetector are 6 and 4 ms, significantly lower than the *τ*_r_/*τ*_d_ of 30/20 ms under −3 V bias and *τ*_r_/*τ*_d_ of 40/154 ms for the NW array photodetector (Figs. 4c and S4b). Under the illumination of 1310-nm laser, the self-powered NIR photodetection behavior of the NW array/NS heterojunction photodetector is further investigated in Figs. 4d and S4c. With increasing incident light intensity from 0.11 to 1.14 mW mm^−2^, the *I*_light_ rises proportionally from 4.7 to 14 pA, leading to a maximum *I*_light_/*I*_dark_ ratio of 182. The NIR photodetection performance of the as-fabricated self-powered NW array/NS heterojunction photodetector is evaluated by calculating the *R* and D*, as shown in Fig. 4e. With the 1310-nm light of 0.11 mW mm^−2^, the R is up to 31 mA W^−1^, while D* is up to 2.21 × 10^8^ Jones. In a word, the splendid photodetection performance confirms the effective strategy of constructing the NW array/NS mixed-dimensional heterojunction for the self-powered NIR photodetection.

### Imaging and Photocommunication of Mixed-dimensional NW Array/NS Heterojunction

**Fig. 5:**
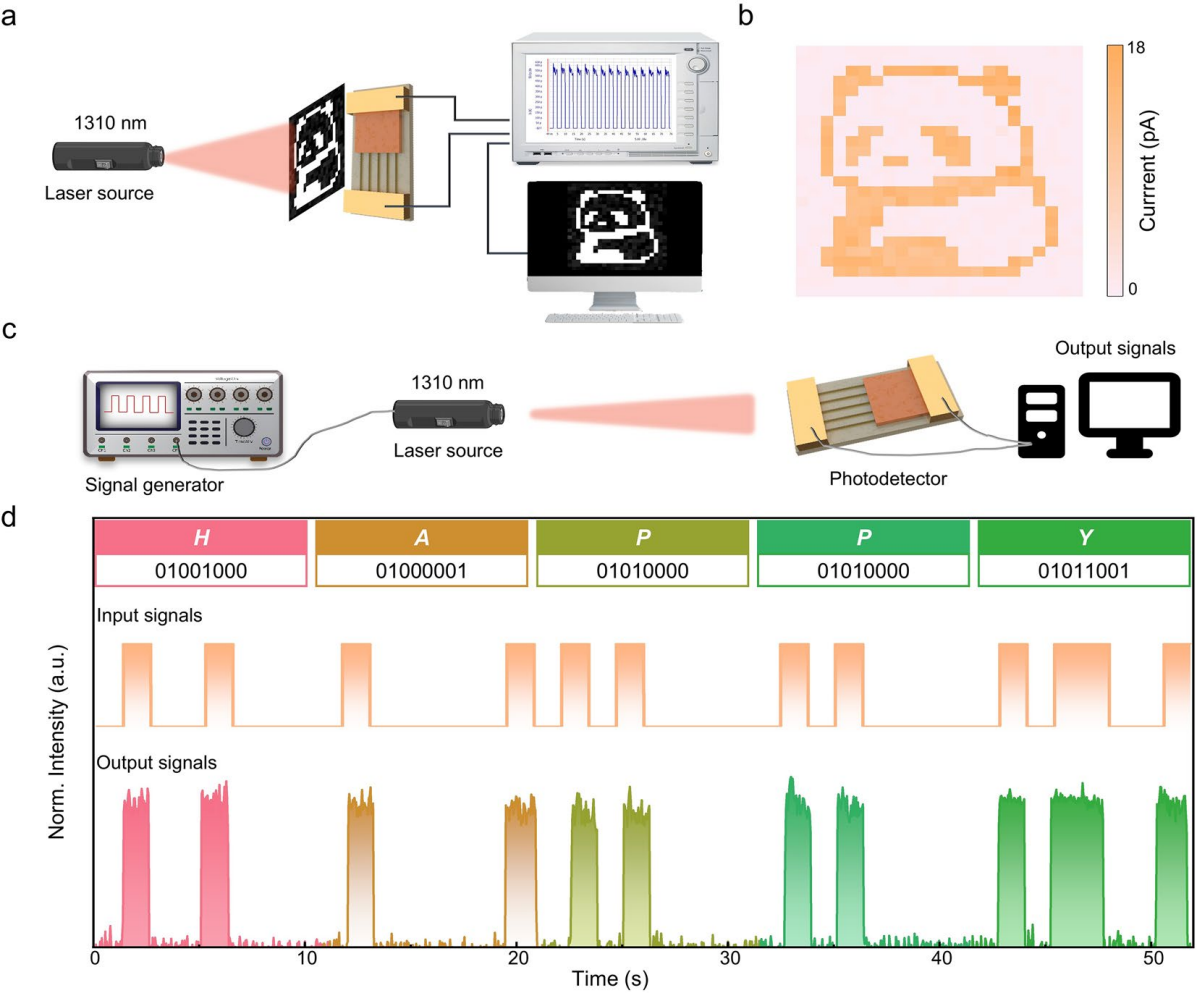
Imaging and optical communication functions of GaSb/Bi_2_O_2_Se mixed-dimensional NW array/NS heterojunction self-powered NIR photodetector. **a** Schematic illustration of the NW array/NS heterojunction photodetector for imaging. **b** Photodetection imaging demonstration of a panda. **c** Schematic diagram of ASCII code signal transportation system. **d** Photocommunication demonstration of ASCII codes of “HAPPY”

The ultrafast photoresponse and superior photosensitivity of as-constructed NW array/NS mixed-dimensional heterojunction self-powered photodetector hold great promise in imaging and photocommunication. Figure 5a provides a conceptual diagram illustrating the imaging principle of the NW array/NS heterojunction photodetector. Figure 5b displays the single-pixel imaging result, showing a clear "panda" image and highlighting the NW array/NS heterojunction photodetector's significant potential for self-powered imaging. Figure 5c depicts a schematic diagram of the NW array/NS mixed-dimensional heterojunction self-powered photodetector integrated into a NIR photocommunication system. A signal generator encodes the ASCII message "HAPPY" into a voltage signal, which is then used to control laser emission. Upon receiving the optical signal, the NW array/NS mixed-dimensional heterojunction self-powered photodetector produces a photocurrent signal corresponding to the ASCII message "HAPPY" as illustrated in Fig. 5d. This photocurrent signal is decoded into the message "HAPPY" effectively demonstrating the feasibility of NIR photocommunication.

## Conclusion

In conclusion, the high-performance self-powered NIR photodetectors are achieved by constructing the mixed-dimensional heterojunction of GaSb NWs and Bi_2_O_2_Se NS, promising the photodetection imaging and photocommunication. Due to the formation of a ~ 140 mV Fermi level difference, the as-fabricated NW/NS and NW array/NS mixed-dimensional heterojunction photodetectors exhibit ultralow *I*_dark_ (0.07 and 0.08 pA) and ultrafast photoresponse (< 2/2 and 6/4 ms). Furthermore, the as-fabricated NW array/NS mixed-dimensional heterojunction self-powered photodetector successfully demonstrated its potential for applications in imaging and photocommunication. Overall, this study promises the as-constructed mixed-dimensional GaSb/Bi_2_O_2_Se NW/NS heterojunction, a novel platform for next-generation high-performance self-powered NIR photodetection, imaging and photocommunication.

## Supplementary Information

Below is the link to the electronic supplementary material.

Supplementary file1 (DOCX 104 kb)
