# Supplementary material for: Mixed-Dimensional Nanowires/Nanosheet Heterojunction of GaSb/Bi2O2Se for Self-Powered Near-Infrared Photodetection and Photocommunication
Source: Nanomicro Lett. 2025 Jun 3;17:284. doi: 10.1007/s40820-025-01793-2 (PMC12133662; doi:10.1007/s40820-025-01793-2)
Supplement: Supplementary file 1 — Supplementary file1 (DOCX 104 kb) [file 40820_2025_1793_MOESM1_ESM.docx]

Supporting Information for

**Mixed-Dimensional Nanowires/Nanosheet Heterojunction of GaSb/Bi_2_O_2_Se for Self-Powered Near-Infrared Photodetection and Photocommunication**

Guangcan Wang^1^, Zixu Sa^1^, Zeqi Zang^1^, Pengsheng Li^1^, Mingxu Wang^1^, Bowen Yang^1^, Xiaoyue Wang^1^, Yanxue Yin^1^, and Zai-xing Yang^1,^*

^1^ School of Physics, State Key Laboratory of Crystal Materials, Shandong University, Jinan 250100, P. R. China

*Corresponding author. E-mail: [zaixyang@sdu.edu.cn](mailto:zaixyang@sdu.edu.cn) (Zai-xing Yang)

**Supplementary Figures and Tables**


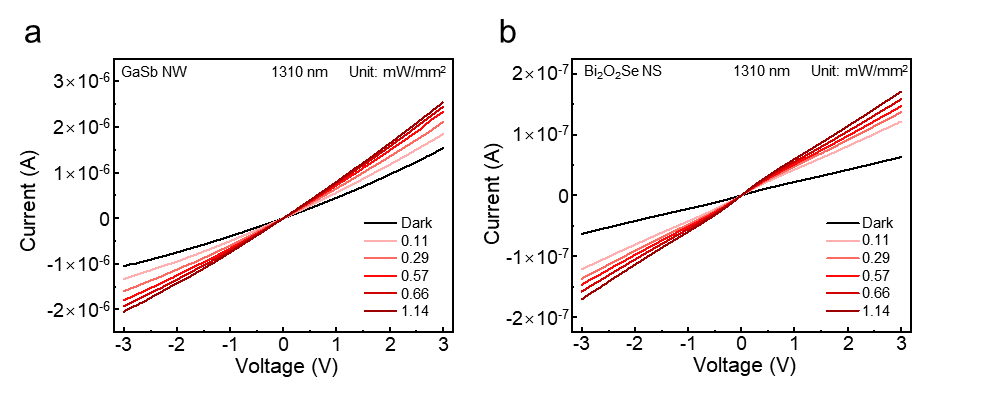


**Fig. S1** I-V characteristics of the as-fabricated GaSb NW (**a**) and Bi_2_O_2_Se NS (**b**) photodetectors under the illumination of 1310 nm laser with various light intensities


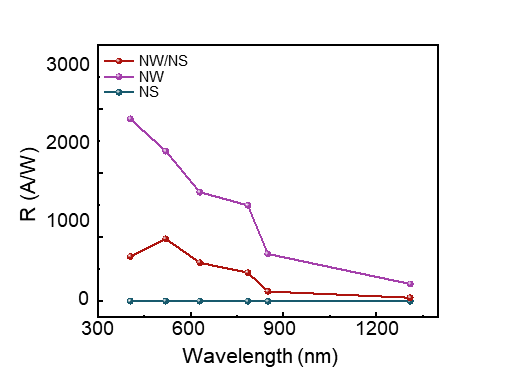


**Fig. S2** Responsivity of the NW, NS and NW/NS mixed-dimensional heterojunction photodetector under the different wavelengths with -3 V bias


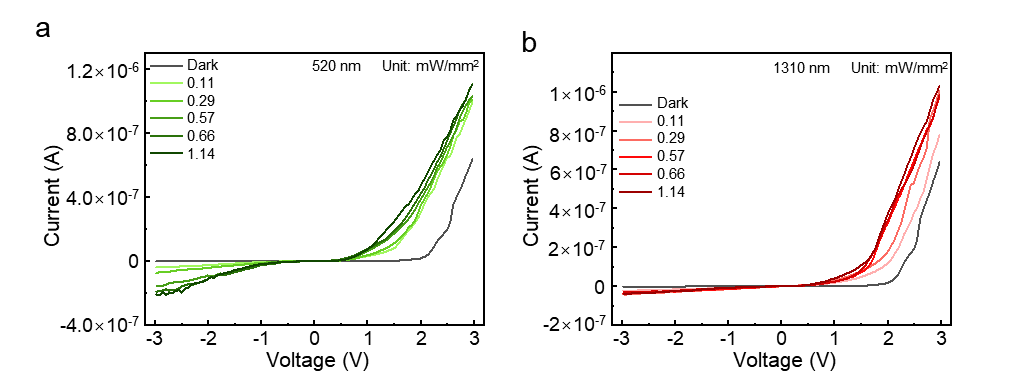


**Fig. S3** Photodetection behavior of NW/NS mixed-dimensional heterojunction photodetector. **a** I-V characteristics of the NW/NS mixed-dimensional heterojunction photodetector under the illumination of 520 nm laser with various light intensities. **b** I-V characteristics of the NW/NS mixed-dimensional heterojunction photodetector under the illumination of 1310 nm laser with various light intensities

**
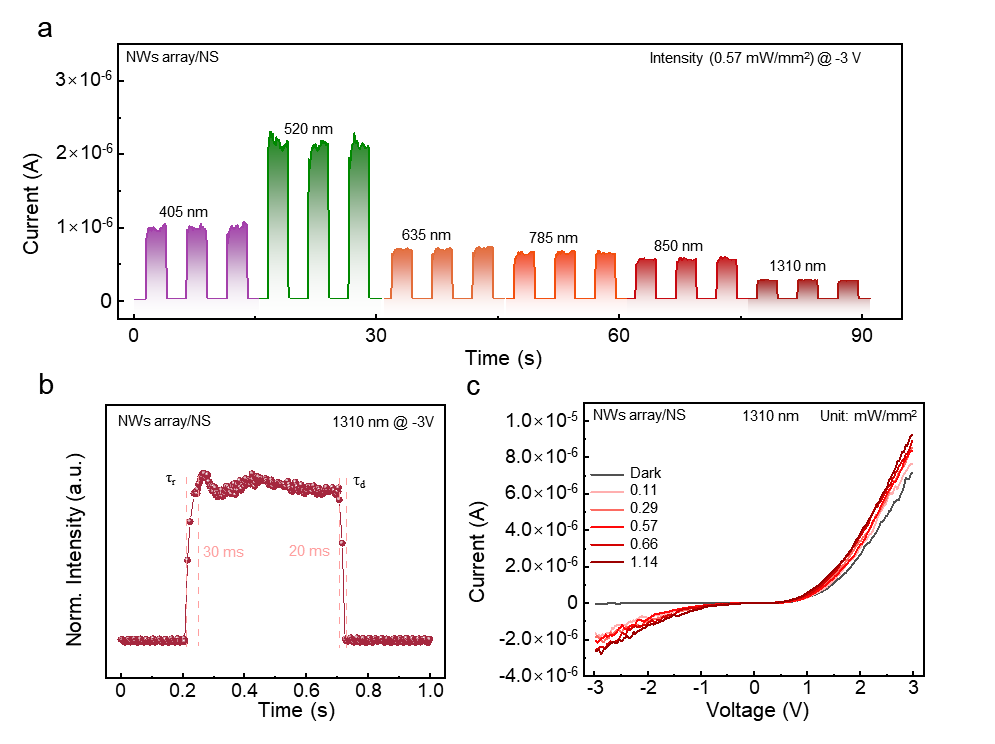
**

**Fig. S4** Photodetection behaviors of the NWs array/NS mixed-dimensional heterojunction. **a** Broad spectrum photodetection performance of the NWs array/NS mixed-dimensional heterojunction photodetector under -3V bias. **b** Response time of the NWs array/NS mixed-dimensional heterojunction photodetector under -3V bias. **c** I-V characterization of the NWs array/NS mixed-dimensional heterojunction photodetector in dark and light conditions

**Table S1** I_dark_ and response time comparison between this work and other photodetectors previously reported in the literatures

| **Marerials** | **Response time** (ms) | **I_dark_** (A) | **References** |
| --- | --- | --- | --- |
| **GaSb** | 11/16 | 4×10^-7^ | [49] |
| **GaSb** | 2/2 | 2×10^-12^ | [13] |
| **GsSb** | 4.5/212 | 3.5×10^-7^ | [48] |
| **Bi_2_O_2_Se** | 39/63 | 1×10^-4^ | [51] |
| **Bi_2_O_2_Se** | 6/20 | 1×10^-9^ | [50] |
| **Bi_2_O_2_Se** | 117/58 | 1×10^-10^ | [52] |
| **Bi_2_O_2_Se NW/MoSe_2_ NS** | 0.38/0.35 | 1×10^-12^ | [53] |
| **Te NW/CdS NS** | 35/45 | 1×10^-8^ | [54] |
| **Te NW/MoSe_2_ NS** | 100/100 | 4×10^-12^ | [56] |
| **Se NW/InSe NS** | 30/37 | 3.8×10^-14^ | [55] |

Notes. The references are in line with Figure 3g.
